# The State Lunatic Asylum Trouble

**Published:** 1887-09

**Authors:** 


### EDITORIAL DEPARTMENT.

F. E. DANIEL, M. D., EDITOR AND PUBLISHER.

COLLABORATORS:

E. J. Doering, M. D., Chicago.
Geo. Cupples, M. D., San Antonio.
Odo Betz, M. D., Germany.
E. J. Beall, M. D., Fort Worth, Texas.
T. C. Osborn, M. D., Cleburne, Texas.
Wm. Penny, M. D., New York.
H. O. Marcy, M. D., Boston.
C. K. Gregg, M. D., Mexico.
R. M. Swearingen, M. D., Austin.
C. H. Wilkinson, M. D., Galveston.
J. W. McLaughlin, M. D., Austin.
R. H. L. Bibb, M. D., Mexico.

### THE STATE LUNATIC ASYLUM TROUBLE.

In our last issue we spoke of the many rumors afloat concerning the management of this institution, and urged, for the sake of the honor and dignity of the medical profession, and of the State, that a searching investigation be had; in order, that if found false these rumors might be forever silenced, and if true, that the proper remedy might be applied, forthwith. The secular press was also outspoken in favor of an investigation. Since our last issue such an investigation has taken place; and as this is *no local matter*, but one in which every physician in Texas, as well as every citizen, feels an interest, and in which the honor and standing not only of a prominent medical man, a member of the Texas State Medical Association are involved, but the dignity of the medical profession and the good name of the State are affected as well, we propose to give the facts in the case; but in the very nature of things we cannot go into details. The rumors which had been flying about like leaves in the wind, finally crystalized, and took shape in the following formulated charges:

IN RE: Charges preferred by Dr. R. S. Johnston against Dr. J S. Dorsett.

*To the Board of Managers of the Texas State Lunatic Asylum:*

Comes now the relator, Dr. R. S. Johnston, and respectfully submits to your consideration the following charges amendatory of and additional to the original list of charges heretofore presented by him against Dr. J. S. Dorsett, to-wit:

First—Prevarication and deception in transactions with Dr. T. J. Bennett, Dr. F. A. Maxwell and Dr. F. E. Daniel, in reference to the retention of the two first named gentlemen in the employ of the asylum.

Second—Prevarication in reference to slapping one Mary Sawyer.

Third—Prevarication to Mrs. Boddy. Prevarication in reference to the infant of a certain colored female patient having been born dead.

Fourth—Drunkenness during his term of superintendency of the asylum.

Fifth—Causing female patients to kiss visitors.

Sixth—Superintendent kissing female patients.

Seventh—Abuse and mistreatment of attendants and patients.

Eighth—Retaining employees who treat patients cruelly.

Ninth—Unjust discrimination between employees in allowing them time for recreation.

Tenth—Not allowing employees to go to town because they would circulate reports as to asylum management.

Eleventh—Unjust discrimsnation against employment of persons from Austin for same reason (sup. 10).

Twelfth—Allowing the most vicious and dangerous patient (Dick Roberts) at the Asylum, and well known to be such, to have an unusual amount of liberty, which resulted in the patients inflicting injuries upon Manuel C. Lopez, from the effects of which he died.

Thirteenth—Permitting garments to be made for employees by asylum seamstresses from goods belonging to the State.

Fourteenth—Discharging patients as restored who are not, and who are immediately reconvicted or returned to the asylum.

Fifteenth—Allowing persons, E. K. Wilson and John C. Nelson, to draw salaries to which they are not entitled.

Sixteenth—Misapplication of asylum funds by paying expenses of attendants to accompany to their homes patients who are discharged as restored.

Seventeenth—Sending patients across the country in an uncovered private conveyance in hot weather, when cheap and comfortable public conveyance was accessible, and the proper means for transporting discharged patients.

Eighteenth—Speaking disrespectfully and evil of the second-assistant physician of the asylum.

Nineteenth—Incompetent in character and profession for the position of superintendent of the Texas State Lunatic Asylum.

The above additional list of charges are true, to the best of my knowledge and belief.

ROBERT S. JOHNSTON.

Subscribed and sworn to before me this 31st of August, A. D. 1887.

R. A. JOHNSTON,
Notary Public.

An additional charge was also filed covering the matter of the altercation between Dr. Dorsett and George Ellis which was tried in a justice's court some time ago.

To all the charges Colonel Robertson entered a verbal denial and plea of not guilty. Before the examination of witnesses began counsel for the prosecution announced that they would abandon charges Nos. 5 and 6, which was so ordered."

The Board of Managers is composed of five prominent citizens of Austin—all honorable and just men, who are duly impressed with the gravity of the charges, and with their responsibility; and they have weighed well, all the evidence, pro and con.

The investigation lasted about ten days, and the Board, on the 14th inst. rendered their verdict to the effect, that eleven of the charges are pronounced by a majority of the board to be proven to their satisfaction. In conformity to a resolution adopted by the Board, the President notified Dr. Dorsett in writing, officially, to that effect, and stated that the board was then ready to receive his resignation. To this notice Dr. Dorsett replied, that to resign, would be an acknowledgment of the truth of the charges, *all of which*, he said, *had been disproved*, and he therefore refused to resign. At this time the matter stands unsettled, but the alternative of the Superintendent's resignation, is said to be that of three (a majority) of the Board.

As the lay-press have connected the local profession of Austin with this investigation in an unenviable light, asserting, in substance, that the prosecution of Dr. Dorset had been instigated by them, and urged on by them, in the interest of the Austin physicians who went out of office with the change of administration, we feel called upon to distinctly declare that there is no foundation for such assumption. It unfortunately became necessary during the investigation, for several of the Austin physicians to testify, and we can bear witness that they did so reluctantly. When the editor of this journal was requested to testify to a conversation had with Dr. Dorset, he rose and said to the Board: "Lest I be misunderstood in connection with this investigation, I wish to state that I am here, *not as a witness*, but as a representative of the medical press, and on special invitation of Dr. Dorset. I know nothing whatever of the management of the Asylum, nor of Dr. Dorset's fitness or unfitness for the position; nor did I know until now (the fourth day) that I was expected to go on the stand. If it is *necessary* to the investigation that I should *repeat a conversation* had with Dr. Dorset, with reference to Drs. Bennet and Maxwell, I can have no objection to doing so." We know that it was also with reluctance that Drs. Swearingen and Denton took the stand; and on behalf of the Austin profession, we utterly repudiate and deny that the prosecution of these charges was instigated or abetted by them.

What will be the final outcome of the trouble remains to be seen, tho' we believe the Board is composed of men who, seeing their duty, and especially having had it distinctly pointed out to them by the daily papers (*Statesman, 15th*), will fearlessly discharge it—*i. e.*, to declare the position of Superintendent vacant—"cause" having been found "good and sufficient." Here it is necessary to explain that only three of the five members of the Board pronounced eleven of the charges sustained, but four of the number concurred in requesting the resignation of the Superintendent.

It is said that there are already nine applicants for the prospective vacancy.

